# Supplementary material for: Corpus callosum anatomical changes in Alzheimer patients and the effect of acetylcholinesterase inhibitors on corpus callosum morphometry
Source: PLoS One. 2022 Jul 27;17(7):e0269082. doi: 10.1371/journal.pone.0269082 (PMC9328497; doi:10.1371/journal.pone.0269082)
Supplement: S1 Table — (DOCX) [file pone.0269082.s001.docx]

**S1 table:** **Clinical characteristics of patients with Alzheimer disease including the disease severity scores before and after treatments**

| **Patient ID number** | **Type of medication** | **Duration (Year)** | **MMSE before treatment** | **MMSE after treatment** | **CDR before treatment** | **CDR after treatment** |
| --- | --- | --- | --- | --- | --- | --- |
| 1 | donepezil | 2 | 14 | 19 | 1 | 1 |
| 2 | donepezil | 2.4 | 11 | 18 | 3 | 2 |
| 3 | donepezil | 3.1 | 19 | 24 | 1 | 0.5 |
| 4 | donepezil | 2.9 | 15 | 22 | 1 | 1 |
| 5 | rivastigmine | 3.4 | 14 | 20 | 1 | 1 |
| 6 | rivastigmine | 2.5 | 14 | 20 | 1 | 1 |
| 7 | rivastigmine | 2.9 | 11 | 19 | 2 | 1 |
| 8 | rivastigmine +donepezil | 2.4 | 5 | 15 | 3 | 2 |
| 9 | rivastigmine | 2.2 | 12 | 15 | 2 | 2 |
| 10 | donepezil | 2.5 | 15 | 21 | 1 | 2 |
| 11 | donepezil | 3.2 | 10 | 14 | 2 | 2 |
| 12 | donepezil | 3.1 | 16 | 25 | 1 | 0.5 |
| 13 | donepezil | 2.9 | 16 | 22 | 2 | 1 |
| 14 | rivastigmine | 2.7 | 13 | 19 | 2 | 1 |
| 15 | donepezil | 3.4 | 15 | 23 | 1 | 0.5 |
| 16 | rivastigmine | 2.8 | 14 | 20 | 2 | 1 |
| 17 | rivastigmine | 3.2 | 10 | 18 | 2 | 2 |
| 18 | donepezil | 2.4 | 20 | 28 | 1 | 0.5 |
| 19 | rivastigmine +donepezil | 3.4 | 7 | 18 | 3 | 2 |
| 20 | donepezil | 3.5 | 19 | 27 | 1 | 0.5 |
| 21 | rivastigmine | 3 | 14 | 25 | 1 | 0.5 |
| 22 | donepezil | 2.9 | 16 | 27 | 1 | 0.5 |
| 23 | donepezil | 2.9 | 16 | 25 | 2 | 1 |
| 24 | rivastigmine | 2.7 | 14 | 19 | 1 | 1 |
| 25 | rivastigmine | 2.9 | 14 | 24 | 1 | 0.5 |
| 26 | rivastigmine +donepezil | 3.8 | 10 | 18 | 3 | 2 |
| 27 | rivastigmine +donepezil | 3.5 | 7 | 16 | 3 | 2 |
| 28 | rivastigmine +donepezil | 2.1 | 11 | 16 | 3 | 2 |
| 29 | donepezil | 2.8 | 9 | 16 | 2 | 2 |
| 30 | donepezil | 2.4 | 16 | 24 | 1 | 0.5 |
| 31 | donepezil | 3.5 | 17 | 25 | 1 | 0.5 |
| 32 | rivastigmine | 3.4 | 8 | 15 | 3 | 2 |
| 33 | donepezil | 3.3 | 17 | 25 | 1 | 0.5 |
| 34 | rivastigmine | 3.5 | 13 | 20 | 1 | 1 |
| 35 | rivastigmine | 2.8 | 11 | 18 | 3 | 2 |
| 36 | donepezil | 2.7 | 14 | 17 | 2 | 2 |
| 37 | rivastigmine | 3.1 | 14 | 22 | 1 | 0.5 |
| 38 | rivastigmine | 3.1 | 10 | 20 | 3 | 2 |
| 39 | rivastigmine | 2.5 | 15 | 21 | 2 | 1 |
| 40 | rivastigmine | 3.4 | 10 | 19 | 3 | 2 |
| 41 | rivastigmine +donepezil | 2.5 | 7 | 17 | 3 | 2 |
| 42 | rivastigmine +donepezil | 3.2 | 6 | 15 | 3 | 2 |
| 43 | rivastigmine +donepezil | 3.1 | 7 | 20 | 3 | 2 |
| 44 | rivastigmine +donepezil | 3.2 | 9 | 21 | 2 | 1 |
| 45 | rivastigmine | 3.5 | 15 | 25 | 1 | 0.5 |
| 46 | rivastigmine | 3 | 9 | 20 | 3 | 2 |
| 47 | donepezil | 3.7 | 16 | 25 | 1 | 0.5 |
| 48 | rivastigmine +donepezil | 2.8 | 7 | 19 | 3 | 2 |
| 49 | rivastigmine +donepezil | 3.3 | 6 | 17 | 3 | 2 |
| 50 | rivastigmine +donepezil | 2.9 | 8 | 20 | 3 | 2 |
| 51 | donepezil | 3.8 | 10 | 19 | 3 | 2 |
| 52 | donepezil | 2.9 | 16 | 23 | 1 | 0.5 |
| 53 | rivastigmine +donepezil | 3.8 | 7 | 21 | 3 | 2 |
| 54 | rivastigmine +donepezil | 3.5 | 9 | 18 | 3 | 2 |
| 55 | rivastigmine +donepezil | 3.5 | 6 | 19 | 3 | 2 |
| 56 | rivastigmine +donepezil | 3.1 | 7 | 20 | 3 | 2 |
| 57 | rivastigmine +donepezil | 2 | 9 | 15 | 3 | 3 |
| 58 | donepezil | 3.6 | 19 | 27 | 1 | 0.5 |
| 59 | donepezil | 3.2 | 13 | 13 | 2 | 1 |
| 60 | rivastigmine | 3 | 9 | 21 | 3 | 2 |
| 61 | rivastigmine +donepezil | 3.7 | 10 | 19 | 2 | 1 |
| 62 | rivastigmine +donepezil | 2.5 | 7 | 20 | 3 | 2 |
| 63 | rivastigmine +donepezil | 3.2 | 9 | 19 | 3 | 1 |
| 64 | rivastigmine | 3.4 | 13 | 24 | 2 | 1 |
| 65 | rivastigmine | 3 | 11 | 22 | 2 | 1 |
| 66 | rivastigmine +donepezil | 2.8 | 10 | 21 | 3 | 1 |
| 67 | rivastigmine +donepezil | 3.5 | 7 | 22 | 3 | 1 |
| 68 | rivastigmine +donepezil | 2.3 | 10 | 14 | 3 | 3 |
| 69 | donepezil | 3.6 | 17 | 26 | 1 | 0.5 |
| 70 | rivastigmine | 3.6 | 12 | 19 | 2 | 1 |
