# Supplementary material for: Corpus callosum anatomical changes in Alzheimer patients and the effect of acetylcholinesterase inhibitors on corpus callosum morphometry
Source: PLoS One. 2022 Jul 27;17(7):e0269082. doi: 10.1371/journal.pone.0269082 (PMC9328497; doi:10.1371/journal.pone.0269082)
Supplement: Attachment — Submitted filename: Response to Reviewers 2.docx [file pone.0269082.s002.docx]

**Point-by-point response:**

**Your valuable comments, suggestions, and remarks are highly valued and appreciated, and they will be taken into consideration**

**Journal Requirements:**

1. **Please review your reference list to ensure that it is complete and correct. If you have cited papers that have been retracted, please include the rationale for doing so in the manuscript text, or remove these references and replace them with relevant current references. Any changes to the reference list should be mentioned in the rebuttal letter that accompanies your revised manuscript. If you need to cite a retracted article, indicate the article’s retracted status in the References list and also include a citation and full reference for the retraction notice**

**Response:** Dear Editor, we have checked all the references, I would like to assure you that all references are complete and correct, and we do not have any retracted references.

**Reviewer #1**

1. **Authors have written that This retrospective study was approved by the institutional research board. Authors didn't mention about institutional ethical committee approval**

**Response:** We thank the reviewer for his careful evaluation of our manuscript, the ethical approval was claimed from the human ethical committee in the Jordan University of Science and Technology, the ethical approval number was IRB #5/134/2020, this already mentioned and highlighted in the manuscript, we also attached the ethical approval document which is in Arabic and a translated copy in English.

**Reviewer #2**

We would like to thank Prof. Preston E Garraghty for his participation in reviewing and evaluating this paper, which help improving the manuscript, thank you.
